# Immune checkpoint inhibitors targeting PD-1/PD-L1 in the treatment of human lymphomas

**DOI:** 10.3389/fonc.2024.1420920

**Published:** 2024-07-18

**Authors:** Domenico Ribatti, Gerardo Cazzato, Roberto Tamma, Tiziana Annese, Giuseppe Ingravallo, Giorgina Specchia

**Affiliations:** ^1^ Department of Translational Biomedicine and Neuroscience, University of Bari Medical School, Bari, Italy; ^2^ Department of Precision and Regenerative Medicine and Ionian Area, University of Bari Medical School, Bari, Italy; ^3^ Department of Medicine and Surgery, Libera Università del Mediterraneo (LUM) Giuseppe Degennaro University, Bari, Italy

**Keywords:** Hodgkin lymphoma, immune checkpoint inhibitors, non-Hodgkin lymphoma, therapy, PD-1/PD-L1

## Abstract

Non-Hodgkin lymphomas (NHLs) encompass a diverse group of malignancies arising from B cells, T cells, and natural killer (NK) cells at various stages of differentiation. Conversely, classical Hodgkin lymphomas (cHLs) primarily feature Reed-Sternberg cells (RSCs) amid a background of reactive immune cells. Immunomodulatory pathways, notably the PD-1/PD-L1 axis, play pivotal roles in tumor immune evasion across both NHLs and cHLs. Elevated expression of PD-1 and PD-L1 is observed in a spectrum of lymphomas, influencing prognosis and treatment response. Therapeutically, immune checkpoint inhibitors (ICIs) targeting PD-1/PD-L1 have revolutionized lymphoma management, particularly in relapsed/refractory cases. Nivolumab and pembrolizumab, among others, have demonstrated efficacy in various B-cell lymphomas, with promising outcomes in cHL. Combination strategies incorporating ICIs with conventional chemotherapy or targeted agents show enhanced efficacy and are being explored extensively. In this review we discuss the most important features of the tumor microenvironment of NHLs and cHLs, address the therapeutic approaches with ICIs and try to outline future perspectives.

## 1 Introduction

### 1.1 Histopathological features of human lymphomas and their microenvironment

Non Hodgkin lymphomas (NHLs) derive from B cells, T cells, and natural killer (NK) cells in different stages of their differentiation. NHLs have been classified into more than 30 different histopathologic types, including indolent lymphomas, aggressive lymphomas, and highly aggressive lymphomas. In classical Hodgkin lymphomas (cHLs), Reed-Sternberg cells (RSCs) constitute a small portion of the involved lymph nodes, while most cells are represented by reactive T cells and other immune cells. cHL are approximately 95% of cases and nodular lymphocyte predominant HL (NLPHL) are approximately 5% of cases of HLs. Hodgkin cells are defined as having a single nucleus and they have proliferative potential while RSCs are multinucleated due to incomplete cytokinesis and lack proliferative potential.

Tumor growth, progression, and metastatic capability in NHLs are influenced by different cellular component of tumor microenvironment, including tumor associated macrophages (TAMs), mast cells, T and B cells, myeloid-derived suppressor cells (MDSCs), tumor-associated neutrophils (TANs), natural killer (NK) cells, dendritic cells (DCs). Over 95% of the cells in the tumor microenvironment of HL are non-malignant cells. RSCs are a minority of cells in tumors (approximately 1%) and recruit monocytes and TAMs, regulatory T cells (Treg), helper T cells (Th), mast cells, fibroblasts, eosinophils, neutrophils, and NK cells, which are all involved in immune evasion.

## 2 Immune checkpoint inhibitors

PD-1 is an inhibitory co-receptor expressed on CD8^+^ and CD4^+^ T cells, NK and B cells, and tumor-infiltrating lymphocytes (TILs) (1). On B-cells, PD-1 is markedly regulated by B-cell receptor (BCR) signaling, lipopolysaccharide (LPS), CpG oligodeoxynucleotides, and several proinflammatory cytokines.

PD-1 interacts with PD-L1, expressed on the cell surfaces of activated T, B, and NK cells (2), peripheral tissues and organs, and tumor cells (3), and PD-L2, expressed by macrophages and DCs (4). PD-L1 is expressed on endothelium in different tumors and correlated with activation of immune cells and a poor prognosis (5). Increased T-cell infiltration observed after anti-angiogenic treatment is associated with enhanced tumor PD-L1 expression (6), epigenetic modifications, and drug resistance (7–9). Overexpression of PD-1 and its ligands, PD-L1 and PD-L2, by malignant neoplastic cells, allows the ligation of PD-1 on T-cells and the consequent induction of T-cell “exhaustion”. In this context, the malignant cells escape from the antitumor immune response (10). Other molecules have been investigated in the microenvironment of HLs, such as lymphocyte-activation gene 3 (LAG-3) and T-cell immunoglobulin and mucin-domain containing proteins 3 (TIM-3) and it was demonstrated that in all cases of HLs there is immune-expression of these pathways, that could be targeted in future clinical trials, particularly, in cases of relapsed/refractory HL. (11).

Immune checkpoint inhibitors (ICIs) have been approved for use in different solid tumors, including metastatic melanoma, advanced non-small cell lung cancer, metastatic renal cell carcinoma, metastatic bladder cancer, advanced head and neck cancer, and hematological tumors, including lymphomas.

To date, 12 antibodies targeting PD-1 and 5 antibodies targeting PD-L1 have been approved by regulatory agencies worldwide. The approved anti-PD-1 and anti-PD-L1 antibodies blocking the axis PD-1 and PD-L1/PD-L2 have confirmed the immune system’s role in mediating the antitumor response, leading T cells to kill tumor cells (12). Different strategies of combining anti-PD1/PDL1 and other immunological agents, chemotherapy, or target molecules have been investigated. The combination of different immunotherapies, targeting distinctive immune checkpoints might be more effective than monotherapy. Indeed, the anti-PD-1/PD-L1 in combination with anti-cytotoxic T-lymphocyte associated protein 4 (CTLA-4), improved objective response rates despite a high rate of toxicities. Thus, ipilimumab, the first new-generation immune checkpoint inhibitor agent approved in monotherapy by the Food and Drug Administration (FDA) in 2011 for the treatment of patients with advanced or metastatic melanoma (13), can be used in combination with nivolumab in advanced melanoma, renal cell carcinoma (RCC), microsatellite instable colorectal cancer, hepatocellular carcinoma (HCC), malignant pleural mesothelioma (14).

Different therapeutic strategies to block PD-1/PD-L1 interaction are under clinical development to prevent PD-1-mediated attenuation of T cell receptor (TCR) signaling, allowing for activity restoration of exhausted CD8^+^ T-cells. In addition, a co-stimulatory signal through B7 protein is required for target-cell lysis and effector cell responses. B7 protein on activated antigen-presenting cells (APCs) can pair with either a CD28 on the surface of a T cell to produce a co-stimulatory signal to enhance the activity of TCR signal and T cell activation, or it can pair with T lymphocyte-associated protein-4 to produce an inhibitory signal to keep the T cell in the inactive state. CTLA-4 inhibition by monoclonal antibodies may induce tumor rejection through direct blockade of CTLA-4 competition for CD-80 (B7-1) and CD-86 (B7-2) ligands, which enhances CD28 co-stimulation. Alternative immune checkpoint molecules expressed on tumor cells or immune cells in the tumor microenvironment can be simultaneously modulated to restore an effective anti-lymphoma immune response.

## 3 PD-1/PDL-1 expression in human lymphomas

**Figure 1:**
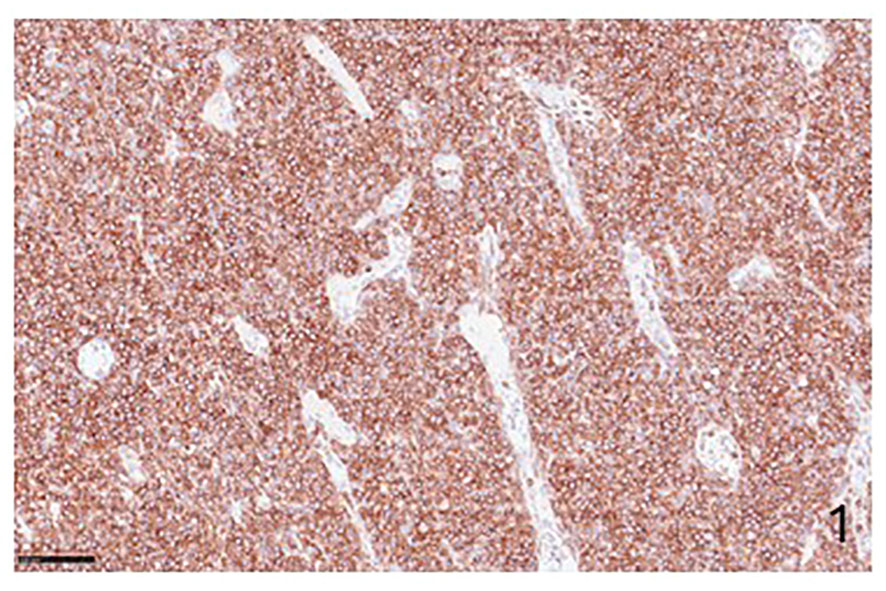
Immunohistochemical preparation showing an example of NHLs with PD-1-positive cells (Immunohistochemistry for anti-PD1, Original Magnification 20x).

**Figure 2:**
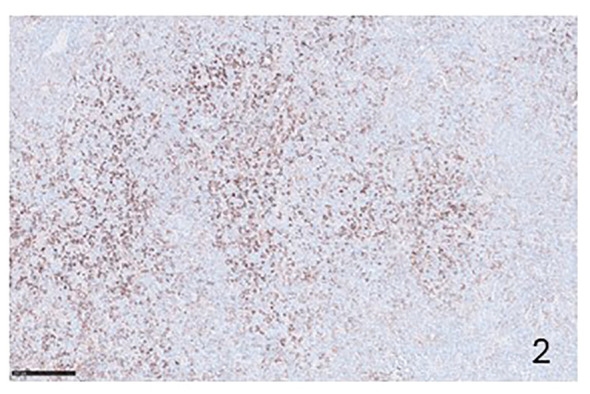
Immunohistochemical preparation showing PD-L1 positive lymphocytes in a case of T-peripheral NHL (Immunohistochemistry for anti PD-L1, Original Magnification 20x).

**Figure 3:**
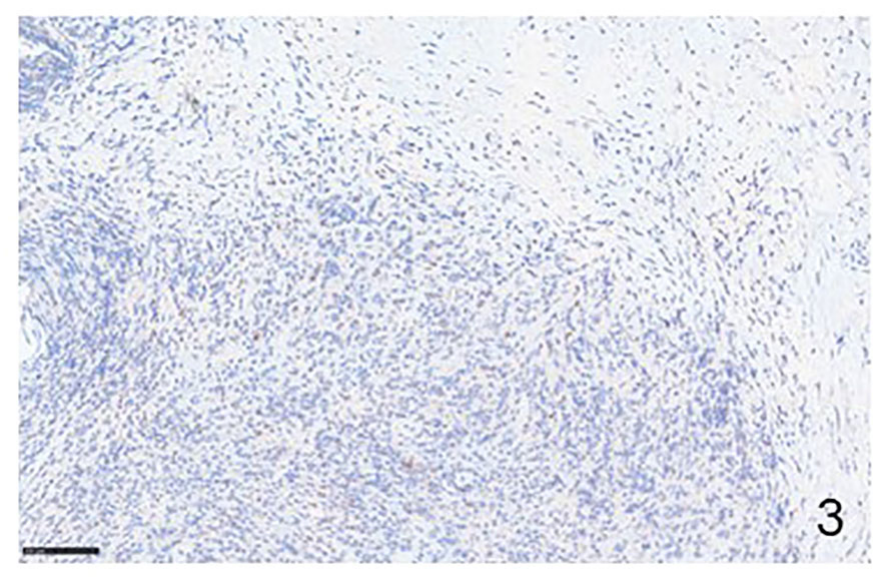
Immunohistochemical example of HLs, classical-type, sclero-nodular, showing some lymphocytes positive for PD-1 (Immunohistochemistry for anti-PD1, Original Magnification 20x).

**Figure 4:**
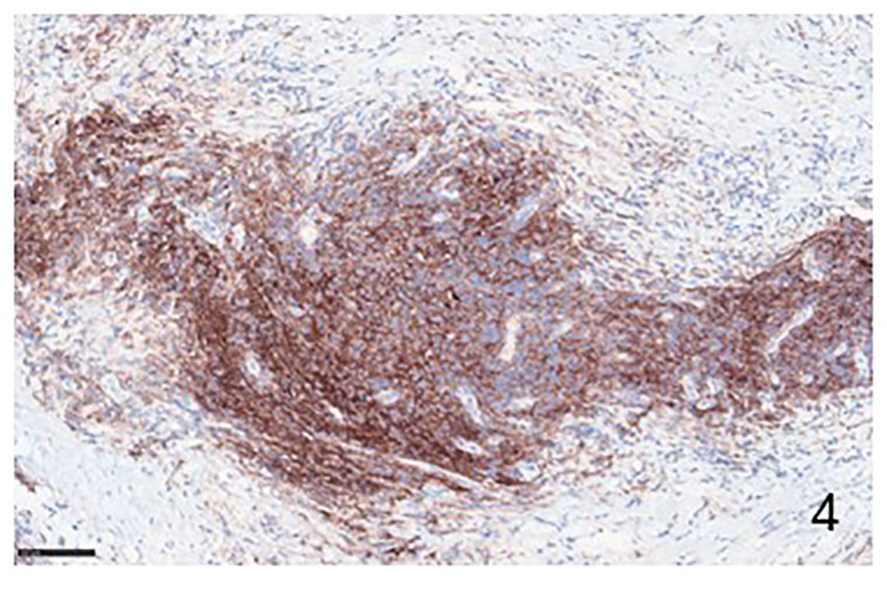
Immunohistochemical example of HLs, classical-type, sclero-nodular, immunolabeled with anti-PD-L1 antibody, showing many positive PD-L1 lymphocytes (Immunohistochemistry for anti PD-L1, Original Magnification 20x).

PD-1 and PDL-1 are expresses in both NHLs and cHLs (**Figures 1**–**4**). In NHLs B cell lymphomas, the highest level of PD-L1 expression has been observed in diffuse large B cell lymphoma (DLBCL), followed by small lymphocytic lymphoma (SLL), mantle cell lymphoma (MLC), while follicular lymphoma (FL) had the lowest PD-L1 expression level (15, 16). In DLBCL, PD-L1 is expressed by the nonmalignant compartment in 26% to 75% of the cases (17). PD-L1 was expressed in about 60% of cases with an average of 20% of patients having a PD-L1/PD-L2 genetic expression in Epstein Barr Virus (EBV)-positive lymphoma (18). EBV infection has been correlated with a much higher PD-L1 expression in DLBCL tumors and a poorer outcome has been reported in cases with PD-L1^+^ macrophages (19). PD-1 expression was detected in 39.5–68.6% of DLBCL cases (20), supporting the notion that a high number of PD-1^+^ TILs are associated with favorable clinical features and prognosis (21). 10.5% of DLBCL samples expression of PD-L1 and PD-1 is associated with poor overall survival (22). Approximately 30-80% of patients with primary mediastinal B cell lymphoma (PMBCL) have PD-L1 overexpression (23). In primary central nervous system lymphoma (PCNSL) and testicular lymphoma it has been demonstrated a high PD-L1 and PD-L2 expression through amplification 9p24.1 (24). In SLL/chronic lymphocytic leukemia (CLL), PD-L1/PD-1 expression ranges between 10-90% (25, 26). FL tumor cells are largely negative for PD-L1 and PD-L2 (27), and the TILs are characterized by high PD-1 expression and suppressed cytokine signaling (28). Several studies have shown that PD-L1 expression is low or absent in MCL (17). In contrast, others have shown a constitutive expression of PD-L1 on tumor cells in both cell lines and primary patient samples (29).

Upregulation and activation of the PD-1/PD-L1 and PD-2/PD-L2 pathways is a typical characteristic of HL (30). In 70–87% of cHL patients, PD-L1 is detected on the surface of both RSCs cells and TAMs (17). It is associated with worse event-free survival (EFS) and shorter progression-free survival (PFS) (31). This overexpression can be consequent to EBV infection (32). EBV infection in cHL increases PD-L1 expression (33). Increased PDL-1 expression by TAMs following interferon (IFN)-γ signaling is relevant in cHL clinical outcomes due to the close relationship between HRS and PD-1^+^ CD4^+^ T-cells (18, 34).

## 4 Immune checkpoint inhibitors in the treatment of lymphomas

Two anti-PD-1 antibodies (nivolumab (Opdivo^®^) and pembrolizumab (Keytruda^®^) and three anti-PD-L1 antibodies (durvalumab, atezolizumab, and avelumab) have been approved for the treatment of B cell lymphomas (35). Nivolumab and pembrolizumab, two fully humanized IgG4-kappa-blocking monoclonal antibodies target the PD-1 receptor on human T-cells. The blockade of the PD-1 signaling pathway by nivolumab induces the proliferation of lymphocytes and the release of IFN-γ. Pembrolizumab binds with high affinity to human PD-1, blocking receptor ligation by both PD-L1 and PD-L2 and leading to enhanced T-lymphocyte immune responses in preclinical models of cancer, with the modulation of key interleukin (IL)-2, tumor necrosis factor-alpha (TNF)-α, and IFN-γ (36).

FL and DLBCL presented the highest objective response to therapy with nivolumab, while MCL lacked a response to treatment (37). In DLBCL treatment with nivolumab, a phase I study demonstrated an ORR of 36% (37), and a phase II study with the same treatment an objective response rate (ORR) of 3% (38). Durvalumab administration in combination with rituximab and bendamustine was associated with an ORR of 88.9% in FL, and of 30% in DLBCL (39). Combination therapy with durvalumab and ibrutinib, a BKT inhibitor, was associated with an ORR in MCL (39).

Ansell et al. investigated the efficacy and safety of nivolumab in relapsed/refractory cHL and demonstrated an overall response rate of 87% and a progression-free survival of 86% at 24 weeks (33). In a further study, the combination of nivolumab with doxorubicin, vinblastine, and dacarbazine (AVD) was evaluated in patients with stage III and IV cHL (40). This study again showed that with the use of nivolumab in the frontline setting an objective response rate of 84% with complete remission in 67% of patients was obtained (40). Another trial evaluated nivolumab-AVD versus brentuximab-AVD in the frontline setting in patients 12 years and older who had stage III-IV disease, demonstrating an increase in progression-free survival with nivolumab-AVD group compared to the brentuximab-AVD group (41). Pembrolizumab, a human immunoglobulin G4 monoclonal antibody that blocks the PD-1/PD-L1 and PD-2/PD-L2 pathway, showed positive survival outcomes for patients with refractory/relapsed cHL (42). A further work compared pembrolizumab and brentuximab vedotin in patients with refractory/relapsed cHL, demonstrating that the two-year progression-free survival and safety outcomes favored the use of pembrolizumab (43). Atezolizumab is a humanized immunoglobulin G1 monoclonal antibody that targets PD-L1 and has previously shown antitumor activity in several tumor types. Younes et al. (44) evaluated the safety and efficacy of atezolizumab in combination with R-CHOP in patients with previously untreated DLBCL and demonstrated that the combination improves complete remission rates compared with controls. Tislelizumab, a humanized anti-PD-1 monoclonal antibody, blocks PD-1 with a high specificity and affinity and is another a promising treatment option in cHL. Song et al. (45) showed that tislelizumab demonstrated a favorable safety profile for patients with relapsed/refractory cHL. Otherwise, a favorable clinical activity has been reported when a PD-1 inhibitor was co-administrated with an anti-CD20 monoclonal antibodies (46).

Low efficacy of ICIs monotherapy in most lymphomas includes defects in antigen presentation, non-inflamed tumor microenvironment, immunosuppressive metabolites, and genetic factors. Clinical trials have investigated the efficacy of ICIs in combined-modal strategies with contrasting results. For example, dual checkpoint blockade with anti-PD-1 and CTLA-4 monoclonal antibodies, with or without STAT3 inhibitors, did not show promising clinical activity in DLBCL and FL (47). Major et al. (48) performed a large, retrospective, multicenter study across 15 US cancer centers of patients with aggressive B-cell lymphomas relapsing after or refractory to chimeric antigen receptor (CAR)-T and subsequently received ICI therapy. The results of this study showed that poor outcomes, particularly among patients relapsing early after CAR-T. The Authors concluded that ICI therapy is not an effective salvage strategy for most patients after CAR-T.

## 5 Concluding remarks

The conventional therapies for human lymphomas are chemotherapy, radiotherapy. More recently, immunotherapy has been successfully proposed as a new therapeutic approach for the treatment of these tumors. The tumor microenvironment allows the discovery of targeted therapies and provides data to improve the prediction of tumor progression. PD-1/PD-L1 controls excessive immunity of cytotoxic T cells leading to the failure of T cell immunity. Tumor cells express PD-L1, and as an effect of PD-1/PD-L1 pathway inhibition, T cells become active and exert more pronounced antitumor effects by rescuing exhausted T cells. Antibodies blocking the interaction PD-1/PD-L1 restore the T cell-mediated antitumor immune response. In B-cell lymphoma, the PD-1/PD-L1 blockade therapy exerts positive effects promoting the formation of a “hot” immune-inflamed tumor microenvironment (49). Combination treatment with CAR-T and nivolumab, or PD-1 inhibition with concomitant radiotherapy can then be used (50).

## Author contributions

DR: Writing – original draft, Writing – review & editing. GC: Data curation, Supervision, Writing – review & editing. RT: Supervision, Validation, Writing – review & editing. TA: Supervision, Validation, Writing – review & editing. GI: Supervision, Validation, Writing – review & editing. GS: Funding acquisition, Writing – review & editing.
